# Regional health governance: A suggested agenda for Southern African health diplomacy

**DOI:** 10.1177/1468018115599817

**Published:** 2015-12

**Authors:** Erica Dale Penfold, Pieter Fourie

**Affiliations:** South African Institute of International Affairs, South Africa; University of Stellenbosch, Department of Political Science, South Africa

**Keywords:** Global South, health diplomacy, health governance, regionalism, SADC, social protection

## Abstract

Regional organisations can effectively promote regional health diplomacy and governance through engagement with regional social policy. Regional bodies make decisions about health challenges in the region, for example, the Union of South American Nations (UNASUR) and the World Health Organisation South East Asia Regional Office (WHO-SEARO). The Southern African Development Community (SADC) has a limited health presence as a regional organisation and diplomatic partner in health governance. This article identifies how SADC facilitates and coordinates health policy, arguing that SADC has the potential to promote regional health diplomacy and governance through engagement with regional social policy. The article identifies the role of global health diplomacy and niche diplomacy in health governance. The role of SADC as a regional organisation and the way it functions is then explained, focusing on how SADC engages with health issues in the region. Recommendations are made as to how SADC can play a more decisive role as a regional organisation to implement South–South management of the regional social policy, health governance and health diplomacy agenda.

## Introduction

Regional organisations can effectively promote regional health diplomacy and governance through engagement with regional social policy. The Union of South American Nations (UNASUR) engages with regional health diplomacy (De Lombaerde and Kingah, 2014), as does the World Health Organization (WHO) office in South East Asia (WHO, 2014a).How do we understand the role of the Southern African Development Community (SADC) in Southern Africa?

There is very limited evidence that SADC plays a role in regional health diplomacy and regional health governance through engagement with regional social policy. This role is primarily played by donors partnered with national governments. There is, however, a strong case to make for the more active involvement of SADC in regional health diplomacy and governance, considering its capacity as a regional organisation, its role in the creation of social policy, the presence of member states and member state funding, and its relationship with donors and multilateral organisations in the region.

This article presents new possibilities for the role of SADC in health diplomacy and governance, contributing to existing debates on the role of regional organisations in determining health diplomacy and governance measures. The gaps identified in current literature include a lack of information on what SADC does specifically to advance regional health governance and Southern regional health diplomacy. We argue that SADC can play a more significant role in health diplomacy and governance in the Global South, through engagement with regional social and health policies. The article shows this possibility by first describing the global principles of health diplomacy and niche diplomacy. It further describes what role SADC plays in health governance in the region and finally shows how SADC can play a stronger role in facilitating and coordinating health governance and diplomacy through social policy, based on the potential capacity it has as a regional organisation.

The evidence of the growth of multilateral regional organisations is significant and pertains specifically to international integration and development coordination (Deacon et al., 2010; Riggirozzi and Tussie, 2012; UN University Institute on Comparative Regional Integration Studies [UNU-CRIS], 2008). Regional organisations and regional social policy feature substantially in social policy literature which considers the role of regional organisations as global players and training hubs (Riggirozzi, 2012; Van Langenhove, 2012). Regional organisations contribute significantly to regional social policy and programmes; for example, Latin American regional organisations have contributed significantly to transnational cooperation with regard to health surveillance and labour migration (Deacon et al., 2010; UNU-CRIS, 2008). Regional social policy literature has focused on cross-border redistributive programmes (including employment projects, food programmes and social protection funds), policy harmonisation and provision of services (Deacon et al., 2007: 4; Te Velde, 2006).

Considering the focus on regionalism in developing contexts, there is limited detailed knowledge of regional social policy and health diplomacy outside of the European Union (EU) context and specifically in terms of Southern regionalism in general and Southern African regionalism in particular. In the health context, there is limited knowledge on social policy and a vast amount of information instead on regional economic integration (see Schiff and Winters, 2003; Te Velde, 2006). Policy research on SADC has not specifically examined health issues and the opportunities for such regional policy commitments to be implemented and embedded in domestic social institutions and policy formation (Deacon et al., 2007, 2010; UN Development Programme [UNDP], 2011).

There is a clear need for the paths of regional social policy and health diplomacy to cross. Regional health diplomacy speaks to the need for regional organisations to consider how they engage on issues of health, in the sphere of political diplomacy, but at the same time consider how they engage on existing regional social policies and the creation of new policies which would promote access to healthcare and medicines in the region. The article speaks specifically to how SADC can play more of a role in engaging regional social policy with regard to health governance and health diplomacy.

## The emergence of health diplomacy in foreign policy

The WHO describes the creation of global health diplomacy as an emergent practice that identifies and understands changes that influence global public health, as well as capacity building for WHO member states, to support collective action to mitigate health risks (WHO, 2015). Global health diplomacy has its roots in the creation of the Global Fund to Fight AIDS, Tuberculosis (TB) and Malaria, President’s Emergency Plan for AIDS Relief (PEPFAR), the Global Health and Foreign Policy Initiative and the Oslo Ministerial Declarations (Fourie, 2013).

The Group of Eight (G8) industrialised countries established the Global Fund to Fight AIDS, TB and Malaria in 2002 to increase international funding for intervention to deal with these diseases. Funding was primarily sourced from the United States. An amount of US$15.6 billion was committed by 2008 to AIDS activities in 140 countries (Fourie, 2013; Thörn, 2011). The United States also launched the PEPFAR, a 5-year, US$15 billion initiative to fight HIV/AIDS, mostly in Africa. PEPFAR is arguably the largest intervention ever undertaken by a single country to address a disease. PEPFAR positioned the US government as a major supporter of activities to counter AIDS in the Global South. By 2008, the programme had provided treatment to 2 million people in 15 focus countries, 12 of which were African (PEPFAR, 2009). PEPFAR and the Global Fund are two key examples of global health diplomacy in action.

The practice of global health diplomacy also extends to niche areas for emerging middle-power foreign policy. These areas are useful for health diplomacy, as they can extend middle-power foreign policy presence. The Global Health and Foreign Policy Initiative and the Oslo Ministerial Declarations were presented in September 2006 and March 2007, respectively. The importance of global health is communicated as being ‘the most important and yet still broadly neglected, long-term foreign policy issue of our time…’ (Støre et al., 2007). Health is prioritised as a key element of foreign policy and development in the Oslo Declaration. Priorities outlined in the Oslo Declaration apply to all regions and contexts, including the SADC region.

## Beyond the Oslo Declaration: Health diplomacy in the last 5 years

Seven years after the Oslo Ministerial Declarations, health diplomacy has become critically important for foreign policy, national governments and regional organisations. Health diplomacy ensures the involvement of foreign affairs ministries, because of the relevance of health for the exercise of soft power (or the ability of states to coerce other countries into mutual cooperation or treaties by providing aid for health or access to health programmes), security policy, and trade, environmental and developmental policies. Countries have to address trans-border issues that threaten global stability, including pandemics and climate change. Health is of national and economic interest, reflecting pressures on national sovereignty and global collective action.

A key example of the emergence of new actors and funding in global health is the Bill and Melinda Gates Foundation. By 2007, the Foundation’s budget had surpassed that of the WHO. The Foundation forms part of a new global health governance elite, or the Health-8 (H-8), which includes the WHO, the World Bank, the Global Alliance for Vaccines and Immunisation (GAVI), the United Nations Children’s Fund (UNICEF), the United Nations Population Fund (UNFPA), the Joint United Nations Programme on HIV/AIDS (UNAIDS) and the Global Fund. Global health governance and diplomacy emphasise cultivating unique connections among state and non-state actors acting transnationally. These partnerships reflect how health diplomacy offers ‘new mechanisms to implement ambitious global health initiatives while at the same time securing favourable perceptions in a changing diplomatic space’ (Katz et al., 2011). The Global Health Programme 2012, launched by the Geneva-based Graduate Institute, represents a defining moment in global health diplomacy training. The programme provides courses for diplomats, health attachés and international organisation staff. WHO staff participate in the programme and individual states such as Turkey and China are getting involved with the Institute to host training (Fourie, 2013; Kickbusch and Kokeny, 2013).

Middle powers from the Global South have the power to challenge the status quo (Jordaan, 2003). Countries such as Brazil, Mexico, South Africa and Turkey can potentially change the global health agenda by reforming as opposed to radically changing what already exists. Key examples of where health reform has been successful for a middle power include Canada, Brazil and South Africa. Canada has integrated global health issues into their foreign policies, as part of preparation processes for the World Health Assembly (WHA), the Pan-American Health Organisation (PAHO) and the Summit of the Americas. The Canadian government has also led funding and support for initiatives such as the Framework Convention on Tobacco Control (FCTC), the Canadian Access to Medicines Regime, the Drugs for Neglected Diseases initiative and additional input on the International Health Regulations and maternal and child health (with additional focus on sexual and reproductive rights (the Muskoka initiative) (Canada’s Access to Medicines Regime [CAMR], 2015; Runnels et al., 2014a).

Brazil and South Africa were instrumental in signing of the Global Health and Foreign Policy Initiative as well as the Oslo Declaration. As noted previously, these middle powers played a key role in determining the parameters of global health diplomacy. Considering South Africa’s position in SADC, the region has a powerful place in the Global South to enact these resolutions (Fourie, 2013). Brazil also formally recognised the right to health in the Brazilian constitution and made health a central foreign policy theme. When antiretroviral (ARV) drugs for HIV/AIDS treatment became available in 1996, the Brazilian government pushed for access to ARV drugs for all HIV-positive people in the country. Brazil worked closely with other countries in the Global South, including the Treatment Action Campaign, to grant licences for local generic production of ARV drugs, despite being in breach of the Agreement on Trade-related Intellectual Property Rights (TRIPS). Brazil also participated in the FCTC, providing leadership and support for the initiative (Alcazar, 2008; Fourie, 2013).

SADC, as an emerging regional organisation, should prioritise health as part of the regional policy agenda. We have noted above that donors and international organisations have played a more active role in assisting with healthy policy and programme implementation than SADC does. The article argues that SADC, as a regional organisation, is positioned to be a more integral presence to the creation of regional social policy, in tandem with regional health governance and through additional positioning as a global health diplomatic actor.

## Regional health governance in Southern Africa: The role of SADC

SADC health governance relates to basic principles of SADC’s origins. SADC objectives are intended to ‘promote sustainable and equitable economic growth and socio-economic development’ (SADC, 2014) through regional integration, effective governance and productive systems among the 15 member states.

The predecessor to the Southern African Development Community Conference (SADC), was established in 1980 to promote economic regionalism, development coordination and cooperation (Malecek and Kabát, 2009; Schoeman, 2002). Each member state took responsibility for a particular sector instead of a regional economic development strategy. The ‘sectoral responsibility approach’ resulted in decentralisation of the structure and the creation of the secretariat in Gaborone, Botswana (Ostergaard, 1990; Schoeman, 2002). The SADCC was viewed as a ‘service organisation’ rather than a regional leadership body. This structure made sense politically, however, as each state was enabled to contribute equally to development across the region. SADC (1992) grew out of the SADCC in 1992 and was restructured again in 2001. The SADC Secretariat was established in 2001 to centralise the governance processes of each member state, whereas previously each member state had been responsible for a sector, as noted above. The restructuring process created complications in trying to assign responsibility for sectors to specific programmes housed at the Secretariat (SADC, 2014; Schoeman, 2002).

Pallotti describes SADC as being a development community without a development policy (Pallotti, 2004). Since 1992 and the dissolution of the SADCC, SADC has struggled to find a cohesive approach to regional integration and development. This is exacerbated by the lack of internal and external communication in the organisation. The competition between member states for private sector investment and development interventions, along with internal political struggles and economic imbalances, has resulted in a regional imbalance (African Development Bank, 2014; Pallotti, 2004).

**Figure 1.**
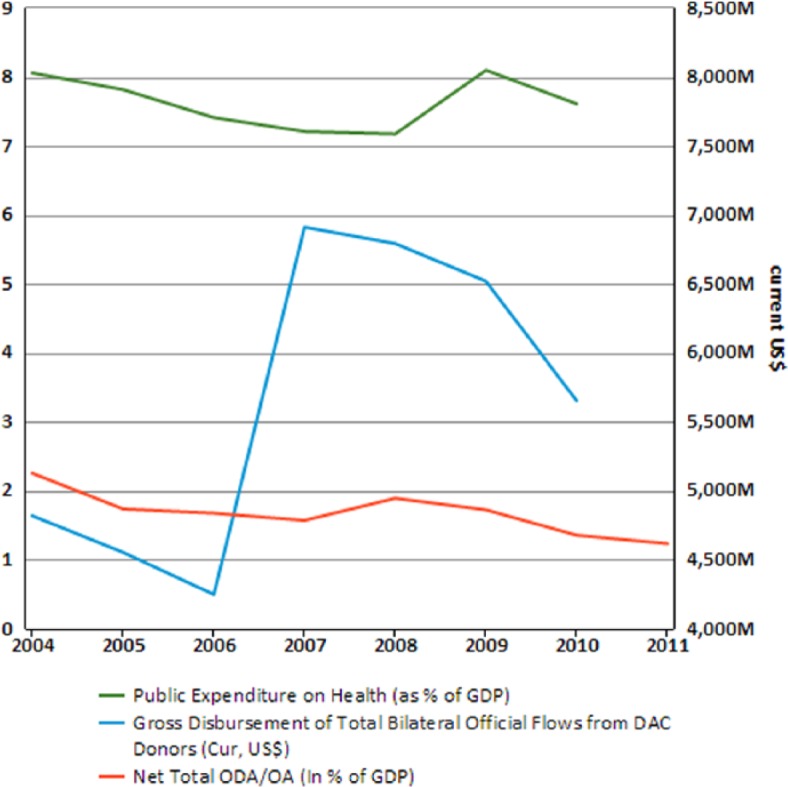
Graph showing the levels of ODA, public expenditure on health and total bilateral flows from donors in Southern Africa. Source: African Development Bank (2014).

Consequently, the political economy of development and the political economy of health in the region are largely influenced by donors from the Global North. This is evident by the large number of aid organisations responsible for coordinating HIV/AIDS plans and projects. Despite this, overseas development assistance (ODA) to the SADC region has declined in the past few years, with member states having to shoulder a large percentage of development funding for their countries. Millennium Development Goal 8 (MDG8) to develop a global partnership for development in the SADC region identifies this decline. ODA has decreased despite the high debt burden in the SADC region. Aid dependence in the region was at the same level in 1999 as it was in 1980. Figure 1 shows the levels of ODA, public expenditure on health and total bilateral flows from donors in Southern Africa (African Development Bank, 2014) The graph indicates a slight increase in public expenditure, with fairly significant decreases in ODA. Despite this, the amount of donor funding is still fairly evident in the region.

SADC member states are responsible for financing their own health budgets and health remains prioritised at a national level, with international support and limited SADC support. There is no specific SADC organisational budget for health policy, as policy is coordinated adopting a bottom-up approach. The money received consists of contributions from SADC member states and donor funding, which is then used by the Secretariat in the development of relevant regional protocols. External finance and foreign aid have encouraged transformation within SADC (with support in particular from the EU, Finland, the United Kingdom, Germany and Switzerland) (Gray, 2013; Tjonneland, 2006). External funding in the SADC region in 2013 amounted to US$67,600,000 for a total of 58 projects (Gray, 2013). The Regional Indicative Strategic Development Plan (RISDP) is said to emulate EU policies of integration and development and is determined by the material dependence of SADC on the EU (Lenz, 2012). As such, donors and governments work together to develop SADC-specific policies and protocols, identified in the next section, which are the foundation for regional health governance in Southern Africa. Perhaps this support is still what SADC requires as a regional organisation. At this stage in SADC’s development, donor aid is essential for sustaining regional health policies and systems strengthening.

## SADC health policies

The SADC Health Protocol forms the cornerstone of regional health governance. It outlines commitments to combating communicable and non-communicable diseases in the SADC region. As such, a number of declarations, plans and strategies have developed from the protocol, detailing specific goals in combating the disease burden in the SADC region. These mechanisms act as frameworks for Southern African regional governance. These plans are detailed in Table 1.

**Table 1.** Southern African Development Community health policies, plans and strategies.

Maseru Declaration on the fight against HIV and AIDS (2003)
SADC Draft Strategic Plan for the Control of Tuberculosis (2007–2015)
SADC HIV and AIDS Strategic Plan (2010–2015)
SADC Malaria Elimination Framework (2010)
SADC Malaria Strategic Framework (2007–2015)
SADC Minimum Standards for the Prevention, Treatment and Management of Tuberculosis (2013–2017)
SADC Pharmaceutical Business Plan (2007– 2013)
SADC Regional Minimum Standards for the prevention of Mother to Child Transmission of HIV (2009)
SADC Regional Minimum Standards for the Prevention, Treatment and Management of Malaria (2010)
SADC Sexual and Reproductive Health Business Plan (2011–2015)
SADC Strategic Framework for Control of Tuberculosis in the SADC Region (2012)
Sexual and Reproductive Health for SADC (2006–2015)
The Draft Declaration on Tuberculosis in the Mining Sector (2012)
The Health Policy Framework (2003)
The Minimum Standards for HIV Testing and Counselling (2009)
The Regional Indicative Strategic Development Plan (2001)
The SADC Minimum Standards for Child and Adolescent HIV, TB and Malaria Continuum of Care (2012)
The SADC Protocol on Health (1999)

The SADC Protocol on Health (signed on 18 August 1999) is the driving document behind health in the SADC region. SADC recognises that ‘a healthy population is a prerequisite for the sustainable human development and increased productivity in a country’ (SADC, 1999). Member states acknowledge that regional cooperation for health is indispensable for controlling communicable and non-communicable diseases and to address common concerns in the region (SADC, 2014). The major policy documents which complement the Health Protocol are the Health Policy Framework and the RISDP.

## Regional governance in the global health sphere – The MDGs and sustainable development goals (SDGs)

The challenges identified speak to global efforts to manage health effectively. The MDGs represent the efforts of the global health diplomatic community to address the health challenges in the Global South. The MDGs, most importantly Goals No. 1, 4, 5 and 6, are seemingly unattainable in the SADC region by 2015. The SDGs, born out of the Rio+20 Conference (United Nations Conference on Sustainable Development), build on the MDGs by focusing on coherent action for economic, political and socially sustainable development. Health is a priority sustainable development theme. Key social issues in the SADC region, which the SDGs intend to address, are high incidences of HIV/AIDS and related diseases and poor health service delivery (Sachs, 2012).

Findings from a study commissioned by the UN, African Union (AU) and the African Development Bank find that the primary use of the SDGs should be to inform their country’s budgets and allocate more resources to SDGs that are not making progress. A secondary use would be to balance economic, social and environmental pillars in policymaking. The study emphasises the need for SADC to draw attention to the SDGs to define national policy priorities, review the influence of national policies and address pressures that lead to unsustainable development and guide development cooperation (Gandure and Kumwenda, 2013).

Effective implementation and achievement of the SDGs will only be advanced by effective coordination of regional organisations, in partnership with national governments and donors. Considering the weak returns of the MDGs in Southern Africa, SADC can, with the correct support and coordination, step up to assist and ensure that national governments can strive to achieve the SDG targets and eventually maintain capacity to do so without foreign support. Resources and political resistance are problematic, but the positives of effective regional health governance outweigh these challenges.

## Five considerations for SADC as a regional policy actor

We posit that SADC has the potential capacity to play more of a role in health diplomacy and social protection in Southern Africa and in the Global South without support from foreign donors. To do so, we argue that if SADC implements the following recommendations for their health agenda, this role could have a greater bearing on regional health diplomacy and governance through regional social policy. To enable SADC to participate as a competent regional power for South–South health diplomacy and act as a social protection champion, we make a number of recommendations which could change how SADC engages with health intra- and inter-regionally.

### Civil society

The SADC Secretariat and health division need to engage further with civil society, non-profit organisations and citizens to ensure that the views of citizens are considered equally. The Oslo Declaration, in identifying health as a ‘pressing foreign policy issue’, calls for recognition of the processes by which the government, multilateral organisations and civil society position health in foreign policy deliberations for health governance (Kickbusch et al., 2007b; Labonté and Gagnon, 2010).

Civil society should be positioned to challenge government decisions and SADC ministerial decisions. Considering the difficulties experienced in democratic governance in Southern Africa, the role of civil society must become a priority in the regional organisation to uphold democratic values and to ensure that the health interests of citizens are met. Labonté and Gagnon indicate that civil society can challenge corporations promoting tobacco, alcohol and obesogenic foods, considering the damage they inflict on societal health. Canada and Brazil have set precedents for this in their work for the FCTC (as noted above). If SADC were to partner strongly with civil society to regulate the introduction and sale of harmful commodities, the distribution of goods and awareness of potential dangers could be better monitored and regulated (Godsäter, 2015; Labonté and Gagnon, 2010).

The increased role of civil society in public health is also attributable to a perceptible weakening of state authority as a consequence of globalisation and the strengthening of transnational corporations (Lee, 2010). Civil society is responsible for promoting ‘transnational’ support for public interests in global health issues. Continued pressure from civil society in the SADC region creates the possibility to allow for public participation in policies and processes, which have traditionally been siloed at a ministerial level (Besharati, 2013; US Agency for International Development [USAID], 2011; Whande et al., 2010). The demand for SADC accountability and responsiveness to citizen outputs makes the work of civil society increasingly more prominent.

The growth of civil society in addressing health issues also represents a shift in development processes. Health development requires careful consideration of the relationship between state and society. The 1978 Alma Ata Declaration represents one of the most significant developments in public health, particularly regarding recognition of citizen participation in health systems as a central tenet to primary healthcare, recognising the role of organised social action for securing health gains. White and Banda provide an exemplary explanation on the role of central civil society organizations (CSOs) in health governance, suggesting that CSOs have a better picture of human and technical needs and capabilities. Partnering with communities has the potential to increase returns on health and agriculture. CSOs can also play a role in disease surveillance. The example provided refers to the monitoring of the severe acute respiratory syndrome (SARS) outbreak since the Chinese government was not willing to disclose the magnitude of the outbreak, leaving the WHO reliant on non-governmental organisation (NGO) and CSO reporting on epidemiological information (White and Banda, 2006). The UN High Level Panel on Civil Society also advocates for increased cooperation between government and civil society. SADC would do very well in taking this engagement to heart to further engage on issues of access to healthcare and medicines.

SADC has the capacity to act as civil society champion in the region. SADC could assist in coordinating civil society platforms by potentially creating a civil society advisory desk, in this case for health organisations. Civil society agendas can be promoted by a specific SADC civil society advisory body to ensure that concerns are addressed at a ministerial level and also to include civil society matters on government agendas. South Africa, as the most powerful SADC state, could lead a civil society initiative to ensure that civil society agendas are addressed at inter-ministerial and government levels. There could alternatively be rotating custody of this responsibility.

### Trade

In order to compete globally and regionally, SADC needs to advance its trade agenda in the region in order to compete with other regional blocs and globally. The SADC Trade Protocol is viewed as ineffective in eliminating non-tariff barriers (NTBs), which negatively influence trade in the region in general but more importantly the movement of pharmaceuticals and health products (Brenton et al., 2005; Kalenga, 2000; KeaneCali and Kennan, 2010).

As a regional trade market, SADC provides opportunities for the promotion of economic development of scale in production and services. Health issues, including communicable disease control, drug purchases, health personnel migration, AIDS response, disease surveillance and supply of resources, are being addressed from national, regional and international perspectives. Despite this, trade restrictions within SADC continue to hinder movement of medical purchases.

The 1996 SADC Trade Protocol established a Free Trade Area in SADC, implemented in 2008. Continued efforts have been made on tariff-reduction schedules, rules on the origins of goods and services, elimination of NTBs, customs harmonisation processes, trade documentation mechanisms and dispute settlement mechanisms. Despite these efforts, national macroeconomic policies have increased inequalities in the region, particularly regarding income, employment and access to education, water and non-health sector inputs into health-related issues. Additional inequalities stem from unequal land ownership, distribution and access to resources. Historical structural adjustment policies and market reforms have also influenced public expenditure, creating high levels of healthcare inequalities. Continued lack of access to healthcare has aggravated the poverty–health nexus, with the state playing a weakened role as health provider (Muroyi et al., 2003).

The SADC Secretariat would benefit from using its allocated health budget for prevention mechanisms, access to primary care infrastructures and services, focused deployment of health personnel to regions in crisis and consideration of redistribution of resources between public and private health sectors. Deacon et al. (2007) argue that a serious consideration of challenges to social policy would include identifying appropriate national social policy responses in order to channel resources to those sectors that need them. This includes identifying reforms for social policy institutions including SADC, which focus on health, education, employment and income maintenance (Deacon et al., 2007).

The SADC Secretariat should channel financial resources into addressing these reforms for the healthcare budget, in line with addressing social policy challenges in the region. This is also emphasised in policy recommendations by the Centre for Disease Control (CDC) with regard to emergency responses to disease control. The CDC suggests countries need to increase the presence of health personnel and systems infrastructure on the ground to mitigate against the threat of rapid disease transmission, as occurred with Ebola (Centre for Disease Control, 2014). This is also suggested by the Institute for Security Studies, who recommend the need for allocated funding in developing countries (Institute for Security Studies, 2014). This pertains specifically to the SADC region and the SADC Secretariat should heed these recommendations. In order to do this, the SADC Secretariat should consider integrating with a regional economic and trade regime and generating further support for states to maintain health systems. This takes into account the tripartite free trade discussions which occurred between SADC, Common Market for Eastern and Southern Africa (COMESA) and the East African Community (EAC) in 2012 and the final communiqué between the first COMESA-EAC-SADC Tripartite Summit in 2008 (SADC, 2012c). Health-promoting trade must facilitate equitable outcomes of private and public health services. This will become imminently more possible when SADC strengthens its negotiating power and capacity in trade frameworks, challenge global trade regimes which exclude Southern interests, build non-economic and trade regimes within the region that drive internal growth, and strengthen global integration terms for the Southern African region.

This is not a very easy process to facilitate, considering the dominance of South Africa in the region. The lack of balance in the region is a cause of concern, considering the tariff advantages for South Africa, cross-border informal trade and limited benefits of intra-regional trade. Other SADC countries actively use NTBs to block trade, as these countries vie for markets. Certain countries fear that their economies will be undermined by cheaper goods in the region and have failed to eliminate NTBs (Keane et al., 2010). The SADC region has been cited as being unprepared for trade liberalisation. The result of applied NTBs is low regional trade and resultant poverty in the trading bloc. NTBs restrict product standards and rules of origin. Borders are still a major obstacle to regional trade (Gillson, 2010).

SADC should consider advancing its trade agenda, focusing on facilitation of technology transfer in the Global South. SADC countries need to consider how to rejuvenate their economies in order to generate revenue to afford innovative ways of addressing health issues using new technology and skills (Fourie, 2013). Regional integration efforts, including COMESA, SADC and Southern African Customs Union (SACU), have all attempted to liberalise trade to increase bilateral trade flows and diversify exports. Continued attempts to harness regional integration will assist in lowering costs for all countries. This will assist increased access to health and pharmaceutical products, considering the current lack of standardisation for these products.

SADC could potentially act as a trade negotiation body between multilateral trade organisations, setting the trade agenda. This is pertinent in the light of the problem with access to pharmaceuticals in the SADC region. There is a lack of harmonisation, regulation and distribution of pharma-products, including the granting and processing of import permits, multiple drug regulatory authorities, offshore drug trials and bioequivalence studies for generics in local populations, registration fees and the World Trade Organization TRIPS agreement on drug access and its implementation in the region (Economic Diplomacy Programme [EDIP], 2014; Kaplan and Laing, 2005; Potsanyane, 2013). SADC can assist by acting as an intermediary and potentially establishing a regional regulatory body to manage and harmonise differing drug registration authorities and regulations in the region.

### Training health professionals

Strengthening SADC’s role in global health diplomacy could also allow for a greater focus on training and retaining health professionals. Of critical concern is the lack of qualified health personnel in the region. A renewed focus on the role of the SADC could address the disproportionate distribution of doctors and nurses in developing regions. This is particularly pertinent in Southern Africa, considering the high percentage of HIV/AIDS and other communicable diseases and the ‘brain drain’ of health personnel to developed nations each year. Alarmingly, on the African continent there are a mere 2.3 health workers per 1000 people. The Americas have a ratio of 24.8 workers per 1000. This imbalance is reflected in the high infection and mortality rates across the continent. SADC can take a proactive role by pushing for renewed focus on health personnel within the region, including providing incentives for professionals to remain in Southern Africa (Gilson and Erasmus, 2005; Samb et al., 2007).

A lack of skilled health professionals results in a non-functional healthcare system, particularly in the public sector, at primary levels of care. Southern African health systems are confronted with scarcity and/or unequal distribution of health personnel. There are inadequate ratios of personnel to population for skilled health personnel and uneven distribution of personnel between public and private sectors, urban and rural areas and tertiary and primary system levels (Fourie, 2013; Gilson and Erasmus, 2005).

The move of healthcare workers from lower to higher income countries (within Southern Africa and the Global North) has resulted in increased international migration, which creates further inequities (Gilson and Erasmus, 2005).

The push and pull factors influencing migration include low remuneration, increased work risks, including HIV/AIDS and TB, unrealistic workloads, inadequate infrastructure and working conditions (Padarath et al., 2007). Country situations, including politically repressive environments, taxation, increasing crime levels and poor service delivery, also play a role in motivating health professionals to leave.

WHO statistics indicate that 31 African states fail to meet the ‘Health for All’ minimum standards of one doctor per 5000 people (USAID, 2003). This is an additional concern, particularly regarding political will, budgeting for healthcare concerns and provision of health personnel. Within Southern Africa, there is wide variation in health personnel per country – for example, more doctors in South Africa than in Lesotho, more nurses in Botswana than the Democratic Republic of Congo (DRC). This issue has called for South–South prioritisation of human resources, staff exchanges in the SADC region and increased dialogue on migration. Specific goals include improving understanding of migration, increased attention to strengthening SADC capacity to manage migration and, of course, increased focus on addressing the issues causing mass migration in the first place.

SADC can act as a regional champion for health personnel and regulation of health personnel in Southern Africa. There is scope for improving personnel trade, employment and movement. The General Agreement on Trade in Services (GATS) provides scope to negotiate multilateral governance protocols. GATS creates a system of international trade rules to ensure equitable treatment of participants, to stimulate the economy of members and to promote trade and development through liberalisation. Using this effectively with SADC at the helm, trade can work to revive the health sector in Southern Africa.

### Training for health diplomats

A SADC-led training programme for health diplomats may also be a hypothetical solution for focused South–South health diplomacy management. Training for SADC country representatives could take place at inter-ministerial meetings and elected diplomatic representatives could benefit from advanced health diplomacy training to ‘prepare professionals’ to manage global health challenges. If such a programme was established and successful, SADC could potentially offer these services to other South–South organisations to learn from other countries’ challenges as well.

The University of Pretoria currently offers the only formal postgraduate programme on diplomacy. This training could be expanded to include health diplomacy and links could be set up between other Southern African university institutions for diplomat training programmes region-wide (Fourie, 2013).

This level of training has been achieved successfully by the Graduate Institute in Geneva and is endorsed by the WHO. Training of health diplomats provides key skills for regional health issues to address challenges faced by developing regions and to achieve the requisite levels of successful health governance and diplomacy. Practical exercises addressed specific global health achievements including the FCTC, Resolutions on Trade and Health and WHA resolutions. This has been successfully attained in other regions, and SADC would benefit from this to bolster the number of qualified health staff to engage on issues of regional governance (Fidler, 2007; Kickbusch et al., 2007a; WHO, 2015). The SADC Secretariat is ideally placed for this role, considering its central location and support role for the 15 member states.

### Participation of South Africa and a PAHO

South Africa is a major player in SADC. South Africa’s status as an emerging middle power is important for the region, considering South Africa’s role in Brazil, Russia, India, China, South Africa (BRICS) and India, Brazil, South Africa (IBSA). South Africa is in a position to put SADC on the map as far as health diplomacy is concerned, considering its importance as a regional organisation in the Global South. South Africa is ideally positioned to create closer ties with Brazil and other emerging middle powers to align common goals for health diplomacy. If South Africa and SADC were able to position the region to reflect positively on middle-power potential, particularly at the WHA, this could also demonstrate middle-power capacity to set the official health diplomacy agenda as opposed to responding to a top-down approach from the Global North (Fourie, 2013; Nauta, 2011).

South Africa, as a regional hegemon, could follow the example of South American states and set up a regional institution focusing on health issues within SADC. In Latin America, the PAHO is a key example of a coordinated regional health presence responsible for technical cooperation among states and mobilisation of partnerships to improve health and quality of life. It is described as being ‘the specialised health agency of the Inter-American System’ (PAHO, 2015) and acts as the WHO Regional Office for the Americas. As a comparison, a pan-African organisation focusing on health could effectively address health challenges, instead of health being relegated to a departmental corridor in the SADC Secretariat. This organisation could be funded by the Chinese government or partner with other middle powers in the Global South.

## Further and future points to consider

Opportunities for SADC are by no means limited to these five recommendations. Additional considerations include SADC involvement in the manufacture of generic medicines, acting as a representative to engage with big pharmaceutical companies. SADC can champion the regional generic drugs manufacturing industry, engage with big pharmaceutical companies globally and advocate for the provision of cheaper medicines for impoverished communities. SADC can learn from the examples set by norm entrepreneurs in India and Brazil in engaging with pharmaceutical companies and reducing the price of patents on medicines (Fourie, 2013). This could strengthen SADC’s position in the region and strengthen the case for reduced prices on medicines.

This could also promote the creation of an integrated health system. SADC has a mandate to harmonise national health policies (SADC, 2014). However, the creation of a SADC-specific regional health organisation with a specific mandate to provide regional healthcare protocols to guide national policy will allow for a coordinated regional response. This is pertinent, considering the mobility of populations in the region, the rapid spread of diseases across mobile corridors and country borders and the lack of coordinated healthcare responses, resulting in disparities in treatment and access to care and medicines. It is important to consider both inter- and intra-regional responses to healthcare in this case.

What is not mentioned in the debate relative to regional health diplomacy and the presence of regional health organisations is the prevalence of regional health organisations, which have the capacity to act as intermediaries for the WHO and the WHA. The inclusion of these organisations and their capacity to shoulder health issues as representatives of the WHO may take some of the burden off the WHO, allowing for specific focus on the social determinants of health per region. SADC could again be instrumental in leading this initiative. Coordinated interaction with donors may allow a regional focus rather than national or multilateral focus. Acting in a coordinated regional manner could advance the way that countries interact with external benefactors.

## Conclusion

Regional social policy creation with specific focus on health governance and health diplomacy needs a strengthened participatory body, to facilitate and coordinate policy development. SADC, as a regional organisation, is ideally positioned to take on the creation and management of regional social policy, regional health governance and diplomacy. However, SADC has experienced significant challenges regarding integration and development and reliance on international partners. In order to shoulder the mantle of regional governance responsibility, SADC needs to make a number of changes, as outlined in the article. If these issues are addressed, SADC can strengthen its potential to manage South–South health diplomacy and regional governance, creating a stronger South–South bloc and redefining the health agenda on a global scale.

SADC has the potential to act as a leader in South–South health regionalism. However, despite the many treaties and protocols in place, SADC is reliant on donors, development aid and external interventions for health governance management. Multilateral health security and diplomacy should encourage active participation from SADC and Southern African ministers to ensure a coordinated approach for focused health initiatives. There is little evidence to suggest that SADC engages with the international health community as a regional diplomatic power, considering SADC’s reliance on the international health community for support. We acknowledge the challenges faced by SADC in the form of political differences, a lack of resources and continued conflict in the region. If governments form partnerships to address these challenges using donor money correctly, the potential to create beneficial change outweighs the problems that the challenges create.
